# Supplementary material for: Multi-Omics Analysis of Multiple Glucose-Sensing Receptor Systems in Yeast
Source: Biomolecules. 2022 Jan 21;12(2):175. doi: 10.3390/biom12020175 (PMC8961648; doi:10.3390/biom12020175)
Supplement: Supplementary file 1 [file biomolecules-12-00175-s001.zip › biomolecules-1557875-supplementary/SupportingInformation/SupportingInformationLegends.pdf]

SUPPORTING INFORMATION

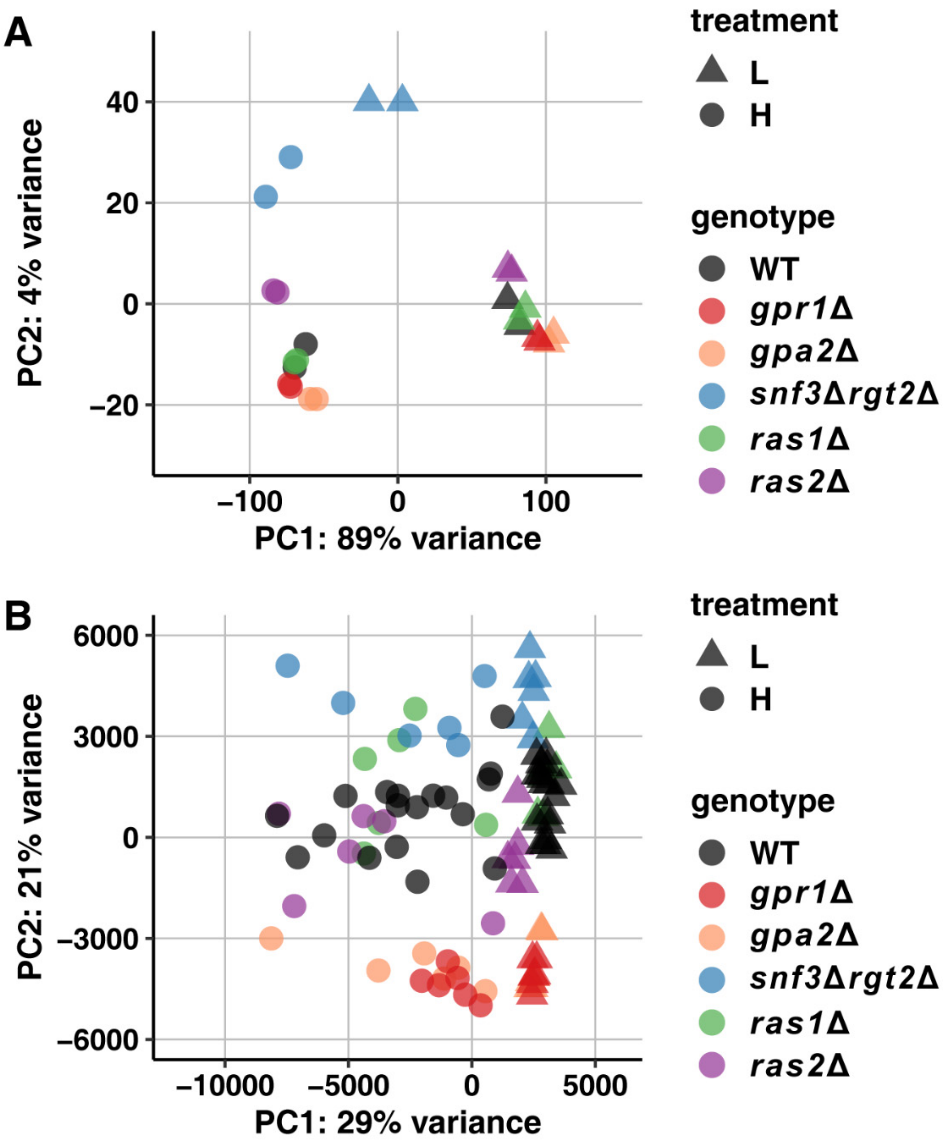

Figure S1. PCA plots. For A) transcriptomics and B) metabolomics, X-axis shows PC1 with the percentage of explained variance and Y-axis shows PC2 with the percentage of explained variance. Data are scaled as detailed in Methods. Wildtype (black), *gpr1*Δ (red), *gpa2*Δ (orange), *snf3*Δ *rgt2*Δ (blue), *ras1*Δ (green), *ras2*Δ (purple). Low glucose (L, 0.05% glucose)-triangles, high glucose (H, 2% glucose)-circles.

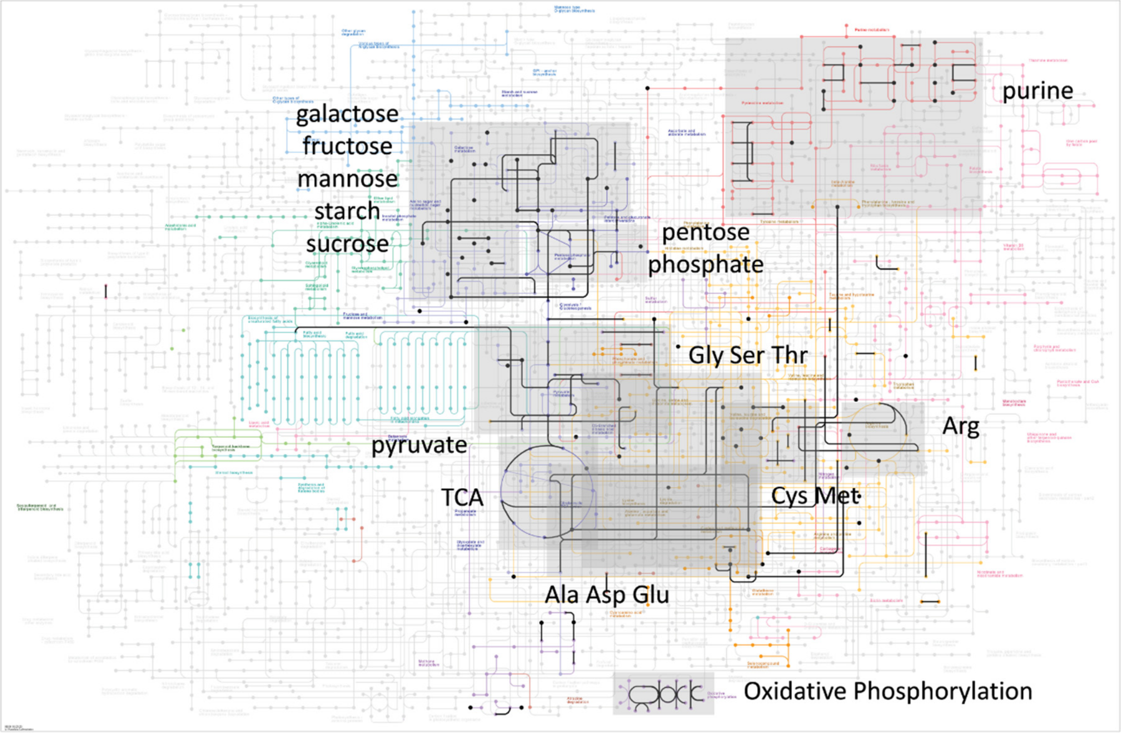

Figure S2. Overview of DEGs and SPMs regulated by *GPR1* projected on KEGG Metabolic Pathway. The map is color coded to delineate carbohydrate metabolism (blue), glycan biosynthesis and metabolism (cyan), amino acid metabolism (yellow), nucleotide metabolism (red), lipid metabolism (teal), metabolism of cofactors and vitamins (pink). Highlighted are the DEGs (black lines) and SPMs (black dots) for *gpr1*Δ integration analysis and gray boxes are used to delineate clusters associated with a specific pathway.

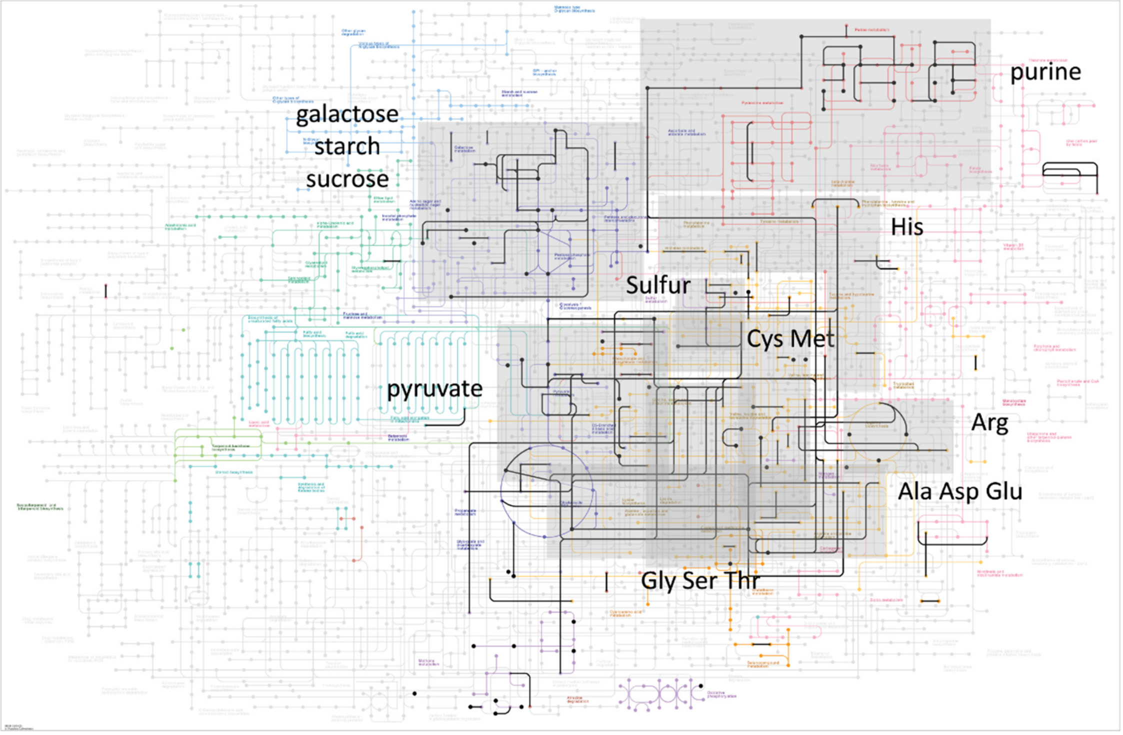

Figure S3. Overview of DEGs and SPMs regulated by *SNF3* and *RGT2* projected on KEGG Metabolic Pathway. The map is color coded as in S2 Fig. Highlighted are the DEGs (black lines) and SPMs (black dots) for *snf3*Δ *rgt2*Δ integration analysis and gray boxes are used to delineate clusters associated with a specific pathway.

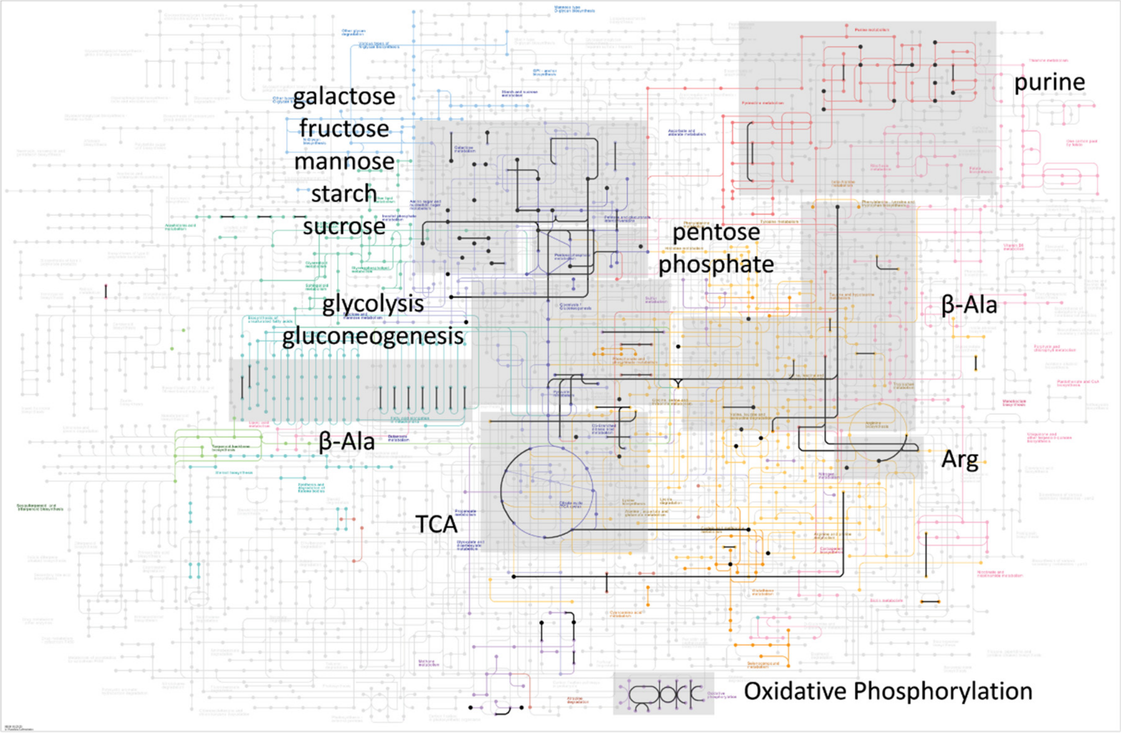

Figure S4. Overview of DEGs and SPMs regulated by *GPA2* projected on KEGG Metabolic Pathway. The map is color coded as in S2 Fig. Highlighted are the DEGs (black lines) and SPMs (black dots) for *gpa2*Δ integration analysis and gray boxes are used to delineate clusters associated with a specific pathway.

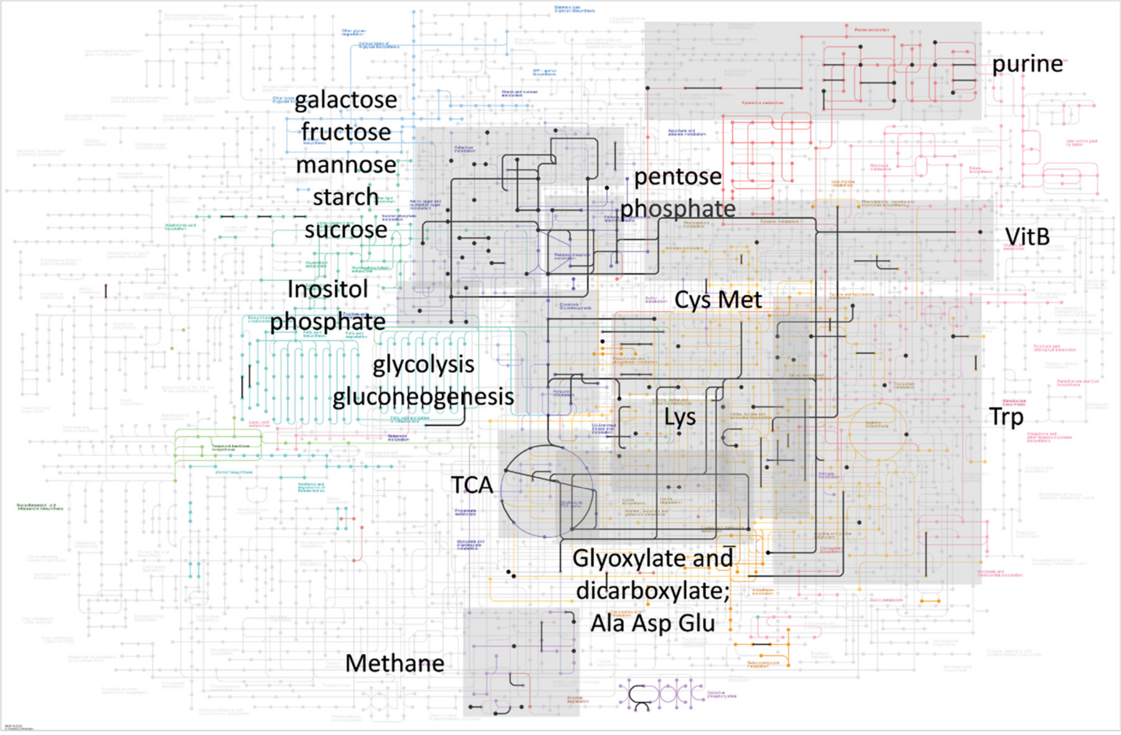

Figure S5. Overview of DEGs and SPMs regulated by *RAS2* projected on KEGG Metabolic Pathway. The map is color coded as in S2 Fig. Highlighted are the DEGs (black lines) and SPMs (black dots) for *ras2*Δ integration analysis and gray boxes are used to delineate clusters associated with a specific pathway.

Table S1. Single-omics analysis results for wildtype between 2% (H) and 0.05% (L) glucose. First block shows GSEA for transcriptomics with adjusted p-value <0.05, arranged in ascending order; second block shows MetaboAnalystR pathway enrichment analysis for metabolomics with combined p-value <0.05 arranged in ascending order. Reproduced from [13].

Table S2. Results and statistics of transcriptomics, metabolomics and multi -omics integration for *gpr1*Δ, each as a separate sheet.

Table S3. Results and statistics of transcriptomics, metabolomics and multi -omics integration for *snf3*Δ *rgt2*Δ, each as a separate sheet.

Table S4. List of DEGs for each subset of the Venn diagram in Fig 2A.

Table S5. List of SPMs for each subset of the Venn diagram in Fig 3A.

Table S6. In house compound identification.

Table S7. Results and statistics of transcriptomics, metabolomics and multi -omics integration for *gpa2*Δ, each as a separate sheet.

Table S8. Results and statistics of transcriptomics, metabolomics and multi -omics integration for *ras2*Δ, each as a separate sheet.

Table S9. List of DEGs for each subset of the Venn diagram in Fig 5A.

Table S10. List of SPMs for each subset of the Venn diagram in Fig 6A.

Table S11. List of DEGs for each subset of the Venn diagram in Fig 8A.

Table S12. List of SPMs for each subset of the Venn diagram in Fig 9A
